# Particle Distribution of Solid Flame Retardants in Infusion Moulded Composites

**DOI:** 10.3390/polym9070250

**Published:** 2017-06-28

**Authors:** Ákos Pomázi, Andrea Toldy

**Affiliations:** Department of Polymer Engineering, Faculty of Mechanical Engineering, Budapest University of Technology and Economics, Műegyetem rkp. 3., H-1111 Budapest, Hungary; pomazia@pt.bme.hu

**Keywords:** fibre-reinforced composite, resin transfer moulding, particle distribution, solid phase additive, flame retardant

## Abstract

Resin transfer moulding (RTM) is commonly used for the production of high-performance fibre-reinforced polymer composites. In numerous application areas, the addition of fillers is necessary to enhance some properties of the polymer matrix or provide it with additional properties, such as flame retardancy. As many of the applied additives are solid phase, the reinforcement layers may filter the solid phase additive particles during RTM, resulting in a non-uniform distribution and uneven performance. Consequently, the proper distribution of the solid phase additives in composites is of key importance. This review primarily aims at facilitating the production of flame retarded structural composites by RTM in cases where the required fire performance can only be achieved with solid additives. First, the parameters influencing the particle distribution, along with the models describing it, are reviewed. Then, analytical methods for determining the particle distribution in composites manufactured by RTM are presented. Finally, the possible solutions to improve the particle distribution of solid phase additives are outlined.

## 1. Introduction

Liquid transfer moulding techniques, including resin transfer moulding (RTM), are increasingly used for the production of fibre-reinforced polymer composites. Their advantages over hand lamination (high productivity at a lower cost, increased fibre-to-resin ratio, controlled dimensional tolerances providing outstanding reproducibility) allow their application in the production of structural components for the automotive and aircraft industries [1,2]. These transport industries have strict safety requirements, which can only be met if the flame retardant (FR) properties of the polymer composite matrix materials are improved. For this purpose, a wide range of FR additives are available, offering the possibility to consider the effect of the FR on the mechanical and thermal properties, environmental issues creating risks for human life and the environment, waste treatment, and recycling [3,4,5]. When the RTM method is applied for the processing of an FR composite, besides these properties, the phase of the additive and its effect on the viscosity of the resin also have to be taken into account. During the RTM process, a liquid resin is injected into a preform consisting of fibre reinforcement, and therefore, solid phase additives may be filtered by the reinforcement layers, leading to the non-uniform distribution of additives, and uneven fire performance as a result. For this reason, liquid-phase flame retardants can be more easily integrated into the RTM process, as only their effect on resin viscosity has to be taken into account. Hand lamination is less sensitive to increased viscosities, and as the reinforcement layers are impregnated one by one, no filtration of the solid additives occurs. On the other hand, hand lamination is less productive and the achievable maximum fibre content is lower. Therefore, RTM is preferred in many industries. In many applications, different additives have to be incorporated into the polymer matrix to enhance existing or provide new properties [6,7,8,9,10]. The most common additive types applied in thermoset polymer composites are:

- formulation additives:
  - air release agents eliminating or preventing the formation of air bubbles (e.g., silicon-containing or silicon-free polymers)
  - dispersants reducing the viscosity and improving the flow behaviour (e.g., various low to high molecular mass polymers)
  - wetting agents and surface modifiers preventing surface defects (e.g., polysiloxanes, polyacrylates)
- performance additives:
  - light stabilizers (e.g., hindered amines)
  - thermal stabilizers (e.g., phenol and phosphite type antioxidants)
  - flame retardants (e.g., metal hydroxides, various halogen- and phosphorus-containing additives)
  - antistatic additives (e.g., quaterner ammonium salts of fatty acids, alkyl sulfonates, alkyl phosphates, alkyl dithiocarbamates, alkyl carboxylates)
  - conductive additives (e.g., quaterner ammonium salts of fatty acids, alkyl sulfonates, alkyl phosphates, alkyl dithiocarbamates, alkyl carboxylates)
- colourants:
  - dyes
  - pigments

The proper dispersion of the additives is a particular challenge when solid-phase additives are added in relatively large amounts to the polymer matrix, as in the case of many common FRs as aluminium trihydroxide, magnesium hydroxide, or ammonium polyphosphate. Accordingly, it is important to explore the possibilities to improve their distribution in composites produced by RTM. Therefore, the present article reviews:

- the parameters influencing particle distribution during RTM,
- the models established for the description of particle distribution in composites prepared by RTM,
- the analytical methods for determining particle distribution in composites manufactured by RTM,
- the possible solutions to improve the particle distribution of solid-phase additives.

As the literature on the distribution of FRs is quite limited, publications related to the distribution of other solid additives are also discussed; with the primary aim of facilitating the production of flame retarded structural composites by RTM in the case of solid FRs.

## 2. Parameters Influencing Particle Distribution during RTM

Liquid moulding techniques, such as resin transfer moulding (RTM), are commonly used to manufacture fibre-reinforced composites. In the RTM process, a fibrous preform (lay-up sequence) is inserted into a rigid half-mould, and the mould is then closed. Following this, resin is injected into the mould with a positive gradient pressure through the gate points, replacing the air entrapped within the preform. At this point, the curing phase begins. After curing, the mould is opened and the composite part is removed. In some cases, a post-curing phase is necessary in order to guarantee a high degree of cross-linking. If solid phase additives are applied in resin formulations, a homogenous and stable suspension of the particles is of paramount importance in these processes [11,12]. To avoid the filtration of the solid particles by the reinforcement, it is important to understand the mechanism of filtering and the process parameters determining its extent. Fundamentally, there are three possibilities in particle-filled resin systems [13]:

1. **No retention:** Particles can flow freely through fibre reinforcement. Uniform particle distribution is possible.
2. **Deep filtration:** Distribution is not uniform. The particles are gathered at the inlet, and the concentration decreases with a higher filtration length.
3. **Cake filtration:** Particles cannot enter the fibre reinforcement. A cake of particles is formed outside the preform entry.

The most relevant process parameters influencing distribution are [1]:

- the pressure applied during RTM,
- the temperature of the resin/suspension,
- the viscosity of the resin/suspension,
- the particle size of the solid additive,
- the pore size of the fibrous media,
- the permeability of the fibre reinforcement,
- the fibre content of the composite,
- the orientation of the fibres,
- the location of the inlet,
- the filling time.

However, the process parameters mentioned above are closely related to each other, e.g., when the temperature increases, the viscosity of the resin decreases, the resin can flow in the cavity more easily, the filling time decreases, and the injection pressure is lower [1]. In the reverse case, when the temperature is lower, the viscosity increases, and so do the filling time and the pressure [1]. There are also connections between the other parameters, which should be taken into consideration.

The ratio between the mean particle diameter and the pore size of the fibrous media is another important aspect to be considered [14,15]. If the resin is filled with large particles (d ≥ 30 µm), volume phenomena prevail over surface phenomena, while for small particles (d~1 µm), surface interactions are dominant. Within these two particle diameters, both volume and surface phenomena can occur [1,14]. Generally, cake filtration occurs when the particle size is larger than the pore size, in which case particles cannot enter the fibre layer and accumulate, forming a cake. When the resin is filled with small particles, the suspension can flow through the fibrous media and only little filtration takes place. For an intermediate ratio of the particle diameter to pore size, deep filtration is the main mechanism [16]. When deep filtration occurs, larger particles are gradually deposited. This can narrow and clog the flow channels within the fibre bundles, which ultimately leads to cake filtration [16].

## 3. Modelling Particle Distribution in RTM

The filtration of solid particles is often modelled with analytical and/or numerical simulations in the literature. The main model for one-dimensional flow and filtration is Darcy’s law (1):

(1) $$\frac{Q}{A}=\frac{K}{\eta }\frac{\Delta p}{L}$$

where *Q* (m^3^/s) is the volumetric flow rate, *A* (m^2^) is the cross-sectional area of the porous channel, *η* (Pas) is the viscosity of the fluid, and Δ*p* (Pa) is the pressure drop measured over a flow distance *L* (m). *K* (m^2^) represents the permeability of the porous media. Lefevre et al. efficiently coupled Darcy’s law (flow model) to a filtration model to describe particle distribution in the cross direction of a composite manufactured by RTM [17,18]. Reia da Costa et al. presented analytical and numerical models of liquid moulding of nanoparticle filled thermosets [19]. These models were also based on Darcy’s law. The authors found a non-linear model incorporating variations in permeability, viscosity, and porosity as a function of local nanoparticle loading. It is obvious that the filtration of the particles by the reinforcement leads to variations in material properties. According to Reia da Costa et al. [19], the narrowing of the reinforcement flow channels caused by the accumulation of nanoparticles results in a reduction of porosity as the resin flow front progresses. They described the relationship between porosity and permeability with the Kozeny-Carman relation, although it should be noted that under the conditions used in their work, the influence on porosity and permeability is negligible due to the small particle retention concentration compared to the volume fraction of the liquid resin. The authors found that the main parameter in this non-linear model was the viscosity of the suspension. Variation in the suspended particle concentration lead to variations in the viscosity. They found a non-linear dependence between the particle concentration and the viscosity. They compared this model to the linear analytical solution in order to validate it and found that these two models are suitable for the simulation of flow and filtration in the liquid moulding of nanoparticle-filled resins. Analytical approximation can be used for short filling lengths, while the non-linear model can be used for infiltration lengths in the meter range. The models developed can serve as a basis for process design or optimization for the production of fibre-reinforced nanoparticle-filled composites where an even distribution of particles is required.

Hwang et al. developed a direct numerical simulation technique to describe particle filtration and flow [20]. They determined hydrodynamic interactions between filler particles and fluid using Stokes-Brinkman coupling to describe the flow in a dual-scale porous media (macro-pores in the intertow space; micro-pores in the intratow space). They modelled fluid flow with Stokes flow, and used the Brinkmann equation as a momentum equation, considering the porous media as a continuum body that can be characterized with its permeability. They found that particle deposition was enhanced in the porous media with a higher permeability. Two mechanisms for this enhancement were reported: one is the penetrating flow into the porous tow and the other is the formation of a downward flow because of the presence of the particles near the porous media. Downward flow occurs in the gap between the particle and porous wall in the direction of particle movement. According to Hwang et al., this flow is responsible for the early deposition of the particle, because this kind of flow is not present in the absence of the particle and yields additional downward penetrating flow through the porous media. In another paper, the fluid-flow-driven motion of particles was studied with Voronoi discretization and minimization of the dissipation rate of energy; they described two-dimensional particle flow and filtration through fibre bundles [21,22]. The results of the simulation were compared to experimental observations. The simulation suggested that clogging occurs if a space is narrower than twice the diameter of the largest particles. Three particle size regions were considered by Frischfelds et al. [21,22]: a medium one with the largest particle size, with approximately half the average spacing between the fibres in the bundles; a second one with twice as large particles as the first set; and the third with half as large particles as the medium set of particles. When they doubled the size of the particles during flow simulation, the overall clogging increased. The particles formed bridges in the channels between fibre bundles. This built up a pressure gradient that pumped particles into the larger openings inside the bundle. Particles enter spaces of the fibre bundle until they get trapped and the further motion of particles at that location is not possible. Experimental results showed a higher accumulation of particles at the front of the fibre bundle, as predicted by the model. Elgafy et al. used a simulation model based on the Eulerian multiphase flow approach to investigate the flow characteristics of carbon nanoparticle filled fluids around a carbon microfibre matrix, and also investigated the interactions between microfibre walls and nanoparticles [23]. They found that the interactions between the microfibre sidewalls and the interfacial fluid layers have a tendency to reduce the flow velocity, causing an attraction of the flow to the microfibre sidewalls. After some time, the flow passages may be blocked. Chohra et al. suggested a so called “sieve mechanism” to describe the macroscopic filtration of the particles: large particles accumulate in front of small pores, causing a concentration distribution within a dual-scale fibrous media [24]; the ratio of particle size to pore size obviously matters. The authors also performed an experimental study on the behaviour of microparticles in dual-scale fibrous media to validate the proposed model. They injected particle-filled resin suspensions along the perpendicular direction of the fibre mats and measured the amount of filtered particles within each layer after rinsing the layers and collecting the particles. Nordlund et al. applied microscopic imaging and microparticle image velocimetry measurements to investigate filtration, using a colourless glycerol/water mixture and fluorescent particles [25]. In the two studies mentioned above, liquid suspensions were investigated instead of fully cured composites. Erdal et al. presented another macroscopic model to investigate particle filtration during RTM, using Darcy’s law and particle retention kinetics [26]. This model was improved by Lefevre et al. to take liquid retention into consideration [17]. The authors found another type of behaviour: the filler concentration along the composite part was merely varying compared to the model presented by Erdal et al. [26]. They found a decrease in filler concentration near the inlet, which was attributed to retention, whereas the accretion near the outlet was assumed to depend on a liquid depletion mechanism in the suspension in the flow front area. These phenomena were taken into account during the improvement of the filtration model. In another paper, Lefevre et al. proposed an enhanced model, which takes into account the changes in viscosity and permeability [18]. According to them, the flow results from a competition between a low permeability and viscosity at the mould inlet and a high permeability and viscosity at the outlet. As the fibrous preform retains fillers, the permeability of the new filter system consisting of the fibrous preform and the retained particles had to be evaluated. Their model also provided clogging detection by monitoring the evolution of the porosity with respect to time: when the porosity was zero, there was no more available volume for the suspension flow.

## 4. Analytical Methods for Determining Particle Distribution in Composites Manufactured by RTM

Several techniques exist for the investigation of particle distribution. However, most of them are based on optical methods such as scanning electron microscopy (SEM) or transmission electron microscopy (TEM). In many research papers, SEM is combined with other techniques, e.g., wavelength dispersive spectrometry (WDS) and electron probe microanalysis (EPMA). A combination of SEM/EPMA/WDS can also be used in fully cured composite parts.

**Figure 1:**
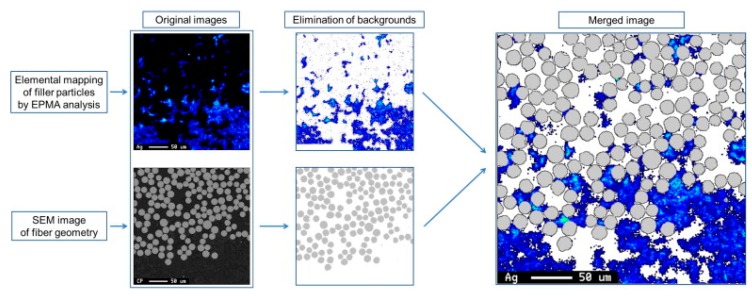
Example of merging the result of the elemental mapping of filler particles by EPMA and the SEM image of the fibre geometry in the cross-sectioned cured composite part including carbon nanotube-silver particles and glass fibres [16].

Yum et al. developed a new methodology for measuring the distribution of particles in fully cured composites via elemental analysis [16]. The matrix resin used was an epoxy system consisting of a bisphenol-A resin with an amine hardener. The fillers were spherical titanium dioxide or carbon nanotube (CNT)-silver particles, and dual-scale fibre mats consisting of E-glass and carbon fibre were used. The width of the flow channels in the intertow region may be up to several hundreds of microns, while in the intratow region, it is a few microns. For qualitative analysis, SEM images were combined with EPMA. This combination, called mapping analysis, provides a visual representation of fibre geometry and the distribution of the particles, as shown in Figure 1. Quantitative analysis is also possible; the filler concentration (particle size range from nanometers to micrometers) on the fully cured composite parts can be measured both in the intertow and intratow region with the use of WDS provided by EPMA. Yum et al. also stated that the degree of dispersion of the filler particles in the resin influences the mechanism of filtration. The resin suspension can be classified into two categories according to the degree of dispersion: well-dispersed particles and clusters of particles [16,27]. It is obvious that the ratio of particle size to pore size determines the filtration mechanism. Clusters are mostly captured by the fibrous preform, and because of their size, cake filtration occurs. Well-dispersed particles—as expected—can flow through the pores of the fibrous media; the main filtration mechanism is deep filtration. With the combination of SEM, EPMA, and WDS, the spatial distribution of the filler particles can be investigated in a fully cured composite part both inside and outside the fibrous media [16].

Fernberg et al. also investigated the mechanisms of particle distribution in infusion moulded composites [28]. They used aluminatrihydroxide (ATH) as a flame retardant; the resin suspension was processed by infusion moulding techniques, including resin transfer moulding. They took into consideration the viscosity of the suspension. They found several models that rely on empirical calibrations [29,30,31], but the following formula had the best correlation with the experimental data (Equation (2)) [30]:

(2) $$\eta =\eta_{S}{\left(1-\frac{\Phi }{\Phi_{m}}\right)}^{-\left[\eta \right]\Phi_{m}}$$

where *η* (Pas) is the viscosity of the suspension, *η_s_* (Pas) is the viscosity of the resin, *Φ* (-) is the phase volume of the filler, *Φ_m_* (-) is the maximum packing fraction of particles, and [*η*] (Pas) is the intrinsic viscosity. The latter is rather a limit of viscosity than an accurate value; it has a physical meaning: it is the viscosity of extremely dilute solutions of a resin in a solvent and is related to the molecular weight of the resin [32]. Equation (2) shows that if *Φ* = *Φ_m_*, then the viscosity of suspension reaches a very high level, mathematically *η* → ∞. If there is no filler in the resin (*Φ* = 0), we cannot talk about resin suspension, only about neat resin; Equation (2) interprets this too: in this case, *η* = *η_s_*. Between these two limits, Equation (2) describes the evolution of suspension viscosity depending on the filler content. Intrinsic viscosity can be determined by the extrapolation of experimental values. Fernberg et al. used an orthophtalic resin designed for vacuum infusion with methyl-ethyl-ketone peroxide hardener, ATH-particles with different sizes, and plain glass fibre weave with an average weight of 816 g/m^2^. Rheological measurements of the resin suspensions with different filler types at various filler mass fractions were carried out. Although there were some discrepancies from the theory expected, Fernberg et al. found that viscosity increases with the decrease of particle size at the same filler volume fraction. At lower concentrations, the suspensions act like Newtonian fluids, but at high concentrations of smaller particles (0.5–5 µm), the suspensions showed a Bingham-type behaviour [18,33,34,35]. This means that no viscous flow occurs under a certain shear stress limit. This Bingham-type behaviour can have a big influence on the mould filling process in liquid infusion moulding processes. The microscopy analysis of the final microstructure of the infusion moulded composites showed that particle-rich areas are always in contact with a fibre bundle; ATH particles can also be observed within the bundles, preferably in resin-rich areas. The authors found that the permeability of the fibre mats does not decrease with flow length, contrary to what they expected. Fernberg et al. expected a decrease in permeability with an increasing flow front position in the case of particle filled resin suspensions because of particle filtration. Interestingly, the permeability did not decrease with flow length; however, after an initial lower permeability, it increased with flow length, and then reached a constant value. According to Fernberg et al. [28], this indicates that particle filtering over time or length only has a minor influence on the overall macroscopic flow behaviour. However, they did not find any sign of systematic differences in the particle distribution along the flow direction. Nevertheless, if the strain rate in a section of the flow channel becomes too low (for example: inadequate flow channel geometry), no flow will occur in that section. In this case, the changes in flow channel geometry can be considered part of the porous structure, decreasing the permeability and providing resistance to flow, thus allowing filtration.

Filtration can be investigated in fully cured composites with a so called “burn-off” technique. In this test, a certain composite part is cut into samples layer by layer and both the matrix and the fibre material of each sample are burned off, so that the mass of the filtered microparticles can be measured in each layer. For this method, both the matrix and the fibrous media should be completely removed by calcination, while filler particles should be non-flammable. Lefevre et al. validated their models with this experimental method [17,18]. In their experiments, the matrix was polyester and the fibre reinforcement was from synthetic polyethylene terephthalate (PET), because PET fibres can be completely removed by burning; the non-flammable filler particles were glass beads. Louis et al. manufactured resin transfer moulded epoxy composites filled with aluminium oxide (Al_2_O_3_) nanoparticles and used aramid fabric as reinforcement [13]. After curing, they examined the local particle distribution by the above-mentioned burn-off method. They measured the resin flow time and particle concentration as process parameters to determine their impact on the final particle distribution in fully-cured composite parts. The burn-off technique is an adaptation of the conventional matrix burn-off (e.g., ASTM D2584 [36]). Because of the aramid reinforcement, they raised the temperature to 1000 °C during the burn-off test, allowing full thermal decomposition. As alumina was used as the filler, only its non-combustible particles remained after burning. They found that 3 h are sufficient at 1000 °C for the burn-off of nominal sample sizes of 0.3 cm × 1.0 cm × 2.0 cm. They also stated that this temperature and method are suitable for composites made with carbon fibres. They observed a non-uniform particle distribution: the highest particle concentration occurred near the inlet and it decreased with an increasing distance from the inlet. They found that with an increasing resin infusion time, the local particle concentration in the laminate increased. Although particle distribution was non-uniform, a desired minimum amount of particles was found across the entire length of the laminate. Louis et al. [13] also investigated the effect of particle concentration in the resin. Their aim was to see whether a lower particle concentration is less sensitive to filtration. Process conditions were controlled, e.g., the same volume of resin and the same overflow conditions (Louis et al. [13] also investigated the effect of fixed resin overflows (5%, 30%, 50%) on the mass percentage of particles retained). However, with a reduced particle concentration, particle filtration still occurred. They concluded that a lower particle concentration does not reduce particle clogging and filtration, and that these are independent of the particle concentration in the resin suspension.

Garay et al. investigated the influence of calcium carbonate on RTM processing and the properties of the moulded composites [37]. Their aim was to investigate resin characteristics, reinforcement permeability, and the mechanical properties of the final composite, while varying the amount of calcium carbonate. Orthophtalic polyester resin was used as the matrix, a combination mat (E-glass fibre mats with polypropylene (PP) flow media core) as reinforcement, and calcium carbonate as the filler, with a mean diameter of 18.22 µm. Particle distribution in the cured composite parts was investigated with the “burn-off” technique according to ASTM D2734 and ASTM D5630. They found that with an increasing calcium carbonate content in the resin, the permeability decreased and a longer time was necessary to fill the mould. The presence of the filler particles between the fibres reduces the porosity of the reinforcement and hinders the flow of resin [25]. Flow in a fibrous media can be classified into two types:

- macro-flow: fluid flows through the spaces between fibre bundles,
- micro-flow: fluid flows within the bundles [38].

The reinforcement used in the experiment was a glass mat with non-continuous, randomly oriented fibre bundles. The presence of the filler was expected to slow down both macro- and micro-flows, although particles were also expected to be found between and within fibre bundles [28]. Garay et al. did not find particle filtration, regardless of the amount of calcium carbonate in the resin suspension, which agrees with the results that Fernberg et al. presented [28]. However, the mass fraction of the glass fibre was not uniform along the mould because of the randomly oriented non-continuous glass fibres and also due to the fact that Garay et al. only used one layer. The results of their study showed that the presence of calcium carbonate impacted the RTM process and the properties of the composite. With the addition of the inorganic filler, the gel temperature decreased and the gel time increased. By increasing the CaCO_3_ content in the resin, the maximum curing temperature decreased and the reactivity of the system decreased. CaCO_3_ has a higher thermal conductivity coefficient (0.24–0.30 W/Km) in comparison to polyester (0.15–0.24 W/Km) and it acts as a kind of a physical reaction inhibitor. The presence of the filler increased the viscosity of the resin suspension according to Equation (2), exceeding the desired value for RTM at higher filler loadings. Furthermore, the addition of CaCO_3_ considerably decreased the permeability of the reinforcement and increased the mould filling time. Although no particle filtration was observed, due to the fibre mat used, these variations in the properties might lead to difficulties in proper RTM processing and manufacturing.

In addition to the different measurement methods mentioned above (SEM, TEM, WDS, EPMA etc.), there are some alternative methods to investigate particle distribution in fibre-reinforced particle-filled composites. Moreover, these alternative techniques have some advantages over methods involving electron microscopy, such as less complicated sample preparation. A very promising non-destructive technique for microstructure characterization is micro X-ray computed tomography (X-ray CT), although it is only suitable for small specimens [39,40]. Confocal fluorescence microscopy (CFM) provides microstructural characterization similar to electron microscopic methods (TEM, SEM), but as its resolution is lower, larger areas can be investigated. For relevant characterization, the material imaged must be autofluorescent or contain fluorescent particles of known properties (size, interactions with the matrix, and the fibres) [41]. Unfortunately, there is a risk that the filtration of these fluorescent particles occurs during RTM processing, which makes confocal fluorescence microscopy unsuitable for structure characterization. Another possible alternative is confocal Raman imaging (CRI). The distribution of the components can be identified with this method at a microscopic scale based on their chemical composition [42,43]. CRI is a combination of confocal microscopy and Raman spectrometry, which analyses the inelastic scattering of coherent monochromatic light produced by a laser. In CRI, Raman spectra are collected with high throughput. The entire Raman spectrum is collected for each pixel, providing a chemical map of the sample [44,45]. Schmidt et al. reported that confocal Raman imaging can be used effectively for the spatial imaging of local phases in a polymeric matrix [46]. Gallos et al. applied CRI to characterize the microstructure of polycaprolactone (PCL) composites reinforced with natural lignocellulosic hemp fibres [47]. Although the samples investigated were prepared with conventional injection moulding for thermoplastics, the paper provides a possible use of the method for the microstructural investigation of polymer composites. They found that CRI is capable of providing information for the entire multicomponent structure. Their results showed that the high sensitivity of the method allows a qualitative evaluation of particle distribution in the composite materials. They claim that CRI can be used for tracking non-fluorescent additives spread in a polymeric matrix over a large surface area. They also investigated the noise that autofluorescence can cause in the Raman signal. For this purpose, they compared CRI with a reference technique based on confocal laser scanning microscopy (CLSM). Their results demonstrated two advantages of CRI against CLSM:

- the ability to characterize small particles that are not visible with CLSM,
- less sample preparation before the measurements.

**Figure 2:**
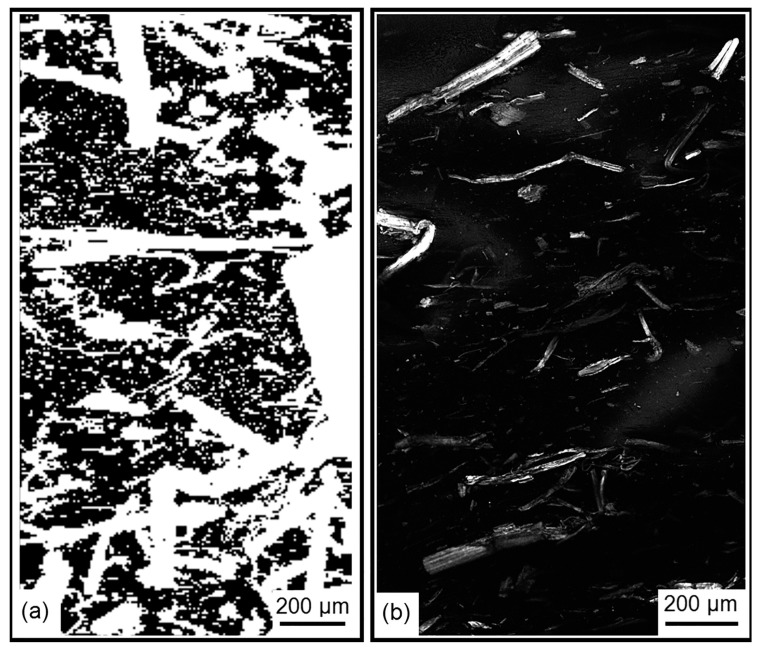
CRI analysis (**a**) and CLSM analysis (**b**) of specimen surfaces of hempf fibre reinforced polycaprolactone composites [47].

Figure 2 shows the comparison between CRI and CLSM analysis of the same sample. It is conspicuous that confocal Raman imaging highlights the presence of small objects, particles, while CLSM does not show these.

**Figure 3:**
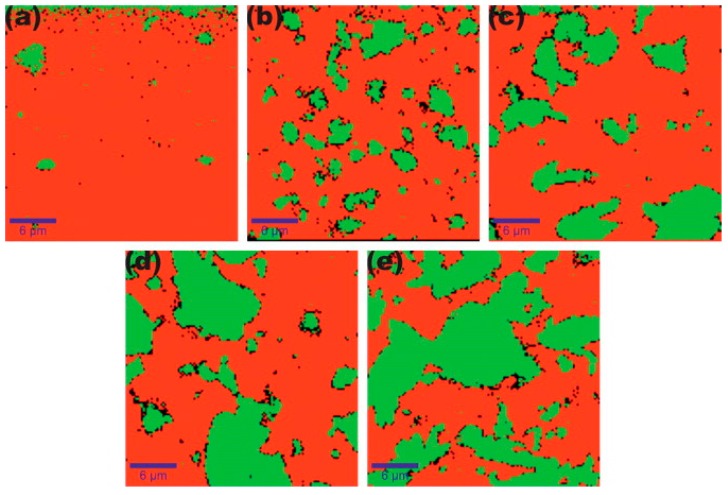
Distribution of graphene (green) in the epoxy matrix (red) obtained by CRI for specimens with a graphene concentration of (**a**) 0.0.5 wt%; (**b**) 0.1 wt%; (**c**) 0.2 wt%; (**d**) 0.3 wt%; (**e**) 0.4 wt% [48].

**Figure 4:**
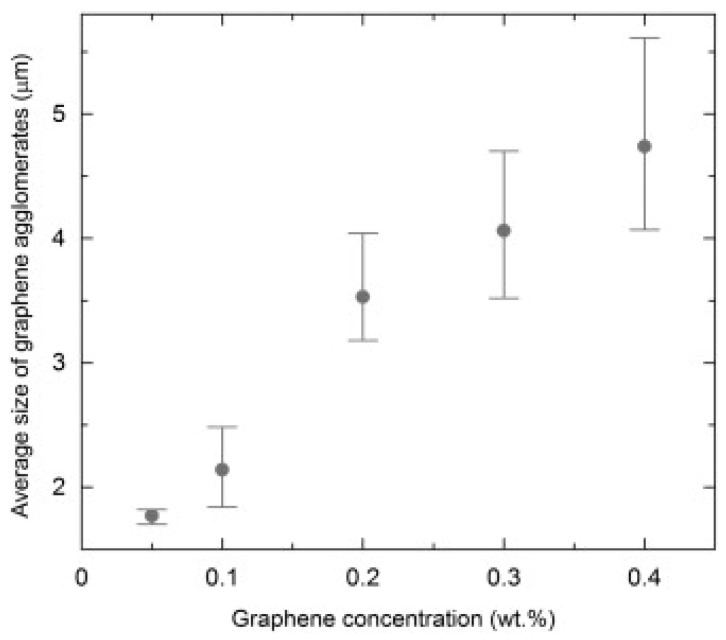
Average size of graphene agglomerates as a function of graphene concentration (Error bars indicate the minimum and maximum values obtained) [48].

Shojaee et al. studied the dispersion and distribution of graphene in an epoxy matrix [48]. To produce phase images, they used confocal Raman imaging. Based on the images taken by CRI, they found not only different distributions, but also different agglomerate sizes in the case of different graphene concentrations, as shown in Figure 3. They stated that the average size of graphene agglomerates increases with an increasing graphene concentration (Figure 4.). Additionally, Shojaee et al. found a jump in the average agglomerate size above a graphene loading of 0.1 wt%. According to them, this jump indicates the optimum graphene concentration for homogenous dispersions. Their findings were in good agreement with the results of another study by Rafiee et al. [49]. Their research focused on graphene/epoxy systems, but obviously confocal Raman imaging can be used to study dispersion and distribution in other additive/polymer systems with appropriate Raman signals. They claimed that CRI provides an accurate, reproducible, and non-destructive way to quantify graphene dispersion in polymer composites. With this method, homogenous distribution can be achieved in composites. Their study suggests that the use of confocal Raman imaging should be taken into consideration as a possible and accurate test method for the investigation of particle distribution in thermosets and their composites.

The main areas of use, along with the advantages and disadvantages of the above mentioned analytical methods for determining particle distribution in composites manufactured by RTM, are summarized in Table 1.

## 5. Possible Solutions

According to the literature, at least one of the filtration mechanisms usually occurs during the RTM processing of particle filled, fibre-reinforced composites. Currently, there is no general solution for avoiding filtration, but some attempts were made to minimize it. For example, Van Velthem et al. used a thermoplastic additive as a carrier for nanofillers in an epoxy-based carbon fabric reinforced composite processed by resin transfer moulding [50]. They investigated dual thermoplastic/nanofiller epoxy resin modification in composites processed by RTM, where the thermoplastic is used as a carrier for the nanoparticles. Their preliminary tests suggested that phenoxy was suitable for this purpose, because the filler they used (carbon nanotubes and nanoclays) could be dispersed particularly well during the melt compounding of the polymer. Neat phenoxy films and particle-filled phenoxy films were manufactured first. These films were then incorporated into the preform between carbon fabric layers before the injection phase of the RTM process. Particle distribution was also investigated in the thermoplastic films by TEM. The distribution of the thermoplastic component and the nanoparticles, as well as their concentration profiles, were evaluated by Raman spectrometry. The TEM images showed that particle distribution in the phenoxy matrix allows its use as a carrier for nanoparticles. During RTM, interdiffusion takes place between epoxy and phenoxy, driven by a thermodynamic force, while nanoparticles in the thermoplastic phase are passively transported. According to these results, the nanoparticles used do not significantly hinder the interdiffusion process and phenoxy distribution in the epoxy resin. Obviously, nanoparticles and thermoplastic distribution are strongly related, and therefore, the local particle concentration depends on the local phenoxy concentration in the resin. Hence, phenoxy seems to be an acceptable soluble thermoplastic carrier for nanoparticles in epoxy resins. Van Velthem et al. also investigated the effect of the temperature of the isothermal step during RTM. They found a strong dependence between the composition gradient and the isotherm temperature: the broadest particle distribution was achieved at the highest temperature. A proper distribution of particles and thermoplastic material in the crosslinked resin improves the properties of the final composite. However, Van Velthem et al. observed significant heterogeneities in the distribution of nanofiller particles. The reasons for this are the following:

- it is impossible to achieve a perfectly homogenous distribution of the thermoplastic carrier without stirring the system;
- some filtering effect by the carbon fabric occurs.

Unfortunately, filtration cannot be avoided with a thermoplastic carrier, due to the “clogging effect” of the particles and the inadequate distribution caused by the carbon fibre fabric and its low permeability. The authors also investigated the morphology of the fully cured composite parts. They found that nanofiller particles end up being surrounded by the cured epoxy phase, even though they were originally dispersed in the thermoplastic phase. The background of this phenomenon might be the preferential adsorption of the epoxy precursors on the nanoparticles during the interdiffusion stage. The aim of the study was to improve the mechanical and fracture properties of the epoxy composites. This may be a good way to toughen composite parts, but the filtering problem of the particles could not be solved efficiently.

## 6. Summary

Fibre-reinforced epoxy resin composites are commonly used in the transport industries. One of the biggest issues to be solved in these systems is the flammability of the organic polymer matrix. Flammability can be decreased and compliance with relevant safety standards can be achieved with the use of flame retardant additives. Many flame retardants are solid, granular materials, which require a uniform distribution in the polymer composite. These composites are often manufactured by resin transfer moulding (RTM), which is a suitable manufacturing method with high productivity required by the industry. If the applied resin contains flame retardant particles, the particles can be filtered through the fibrous media during the injection phase of RTM. Different filtration mechanisms can be observed depending on the ratio of particle size to pore size, the permeability of the fibrous media, and the process parameters of RTM. The factors influencing the mechanism of filtration must be taken into consideration and the relevant parameters should be chosen properly. Unfortunately, filtration usually occurs and cannot be completely avoided. A solution to filtration can only be found if the phenomenon is understood in detail. The mechanism of filtration can be investigated by modelling. The models found in the literature are all based on Darcy’s law. Modelling is useful for understanding; but qualitative and quantitative analysis is also important. For these, there are some common techniques; most of them are based on optical analysis or a combination of methods. Optical microscopy, transmission electron microscopy (TEM), and scanning electron microscopy (SEM) are usually used, often with a combination of wavelength dispersive spectrometry (WDS) or electron probe microanalysis (EPMA). With the above methods, both qualitative and quantitative analysis is possible. Fully cured composite parts can be investigated with other techniques as well, such as the so-called “burn-off” technique (which is an adaptation of the conventional matrix burn-off), confocal fluorescence microscopy, X-ray computed tomography (X-ray CT), or confocal Raman imaging. The latter proved to be an accurate method for studying the particle distribution in thermoplastics, thermosets, and their composites. Though there are attempts to avoid filtration (e.g., the application of a thermoplastic carrier for particles), filtration is still an unsolved issue during the resin transfer moulding of particle-filled fibre-reinforced composites.

## Figures and Tables

**Table 1:** Analytical methods for determining particle distribution in composites manufactured by RTM.

Method	Abbreviation	Main Area of Use	Advantages	Disadvantages
transmission electron microscopy	TEM	fully cured parts	qualitative analysis	sample preparation
scanning electron microscopy	SEM	fully cured parts	qualitative analysis	sample preparation
wavelength dispersive spectrometry	SEM/WDS	fully cured parts	qualitative/quantitative analysis	sample preparation
electron probe microanalysis	SEM/EPMA	fully cured parts	qualitative/quantitative analysis	sample preparation
burn-off	-	fully cured parts	quantitative analysis	total calcination
X-ray computed tomography	X-ray CT	uncured/cured parts	non-destructive	small specimens
confocal fluorescence microscopy	CFM	uncured/cured parts	large experimental areas	lower resolution than SEM, autofluorescence, fluorescent particle needed
confocal laser scanning microscopy	CLSM	uncured/cured parts	more information about fibres than in case of CRI	lower resolution than CRI
confocal Raman imaging	CRI	uncured/cured parts	chemical map, small particles detected	time consuming, fluorescent noise

## Abbreviations

RTM	Resin transfer moulding
FR	Flame retardant
SEM	Scanning electron microscopy
TEM	Transmission electron microscopy
WDS	Wavelength dispersive spectrometry
EPMA	Electron probe microanalysis
CNT	Carbon nanotube
ATH	Aluminatrihydroxide
PET	Polyethylene terephthalate
Al_2_O_3_	Aluminium oxide
PP	Polypropylene
DSC	Differential scanning calorimetry
CaCO_3_	Calcium carbonate
X-ray CT	X-ray computed tomography
CFM	Confocal fluorescence microscopy
CRI	Confocal Raman imaging
PCL	Polycaprolactone
CLSM	Confocal laser scanning microscopy

## References

[1] Laurenzi S., Marchetti M. (2012). Advanced Composite Materials by Resin Transfer Molding for Aerospace Applications. Composites and Their Properties.

[2] Groessing H., Becker D., Kaufmann S., Schledjewski R., Mitschang P. (2015). An evaluation of the reproducibility of capacitive sensor based in-plane permeability measurements: A benchmarking study. Express Polym. Lett..

[3] Hergenrother P.M., Thompson C.M., Smith J.G., Connell J.W., Hinkley J.A., Lyon R.E., Moulton R. (2005). Flame retardant aircraft epoxy resins containing phosphorus. Polymer.

[4] Perret B., Schartel B., Stöβ K., Ciesielski M., Diederichs J., Döring M., Krämer J., Altstädt V. (2011). Novel DOPO-based flame retardants in high-performance carbon fibre epoxy composites for aviation. Eur. Polym. J..

[5] Toldy A., Szolnoki B., Marosi G. (2011). Flame retardancy of fibre-reinforced epoxy resin composites for aerospace applications. Polym. Degrad. Stab..

[6] Geller S., Krafczyk M., To J., Turek S., Hron J. (2006). Benchmark computations based on lattice-Boltzmann, nite element and nite volume methods for laminar ows. Comput. Fluids.

[7] Gibson R.F. (2010). A review of recent research on mechanics of multifunctional composite materials and structures. Compos. Struct..

[8] Toldy A., Niedermann P., Pomázi Á., Marosi G., Szolnoki B. (2017). Flame Retardancy of Carbon Fibre Reinforced Sorbitol Based Bioepoxy Composites with Phosphorus-Containing Additives. Materials.

[9] Molnár K., Szebényi G., Szolnoki B., Marosi G., Vas L.M., Toldy A. (2014). Enhanced conductivity composites for aircraft applications: Carbon nanotube inclusion both in epoxy matrix and in carbonized electrospun nanofibres. Polym. Adv. Technol..

[10] Marosi G., Szolnoki B., Bocz K., Toldy A., Wang D. (2016). Fire retardant recyclable and bio-based polymer composites. Novel Fire Retardant Polymers and Composite Materials: Technological Advances and Commercial Applications.

[11] Fiedler B., Gojny F.H., Wichmann M.H.G., Nolte M.C.M., Schulte K. (2006). Fundamental aspects of nano-reinforced composites. Compos. Sci. Technol..

[12] Thostenson E.T., Ren Z., Chou T.-W. (2001). Advances in the science and technology of carbon nanotubes and their composites: A review. Compos. Sci. Technol..

[13] Louis B.M., Maldonado J., Klunker F., Ermanni P. (2014). Measurement of Nanoparticle Distribution in Composite Laminates Produced by Resin Transfer. Compos. Mater..

[14] Sakthivadivel R. (1969). Clogging of a Granular Porous Medium by Sediment, Report HEL.

[15] Ryan J.N., Elimelech M. (1996). Colloid mobilization and transport in groundwater. Colloids Surf. A Physicochem. Eng. Asp..

[16] Yum S.H., Roh J.U., Park J.M., Park J.K., Kim S.M., Lee W.I. (2013). Assessment of particle distribution in particle-containing composite materials using an electron probe microanalyzer. Compos. Sci. Technol..

[17] Lefevre D., Comas-Cardona S., Binétruy C., Krawczak P. (2007). Modelling the flow of particle-filled resin through a fibrous preform in liquid composite molding technologies. Compos. Part A Appl. Sci. Manuf..

[18] Lefevre D., Comas-Cardona S., Binetruy C., Krawczak P. (2009). Coupling filtration and flow during liquid composite molding: Experimental investigation and simulation. Compos. Sci. Technol..

[19] Reia da Costa E.F., Skordos A.A. (2012). Modelling flow and filtration in liquid composite moulding of nanoparticle loaded thermosets. Compos. Sci. Technol..

[20] Hwang W.R., Advani S.G., Walsh S. (2011). Direct simulations of particle deposition and filtration in dual-scale porous media. Compos. Part A Appl. Sci. Manuf..

[21] Frishfelds V., Lundström T.S. (2011). Modelling of particle deposition during impregnation of dual scale fabrics. Plast. Rubber Compos..

[22] Lundström T., Frishfelds V. (2012). Modeling filtration of particulate flow during impregnation of fabrics. J. Compos. Mater..

[23] Elgafy A., Lafdi K. (2006). Carbon nanoparticle-filled polymer flow in the fabrication of novel fiber composites. Carbon.

[24] Chohra M., Advani S.G., Gokce A., Yarlagadda S. (2006). Modeling of filtration through multiple layers of dual scale fibrous porous media. Polym. Compos..

[25] Nordlund M., Fernberg S.P., Lundström T.S. (2007). Particle deposition mechanisms during processing of advanced composite materials. Compos. Part A Appl. Sci. Manuf..

[26] Erdal M., Guceri S.I., Danforth S.C. (1999). Impregnation molding of particle-filled preceramic polymers: Process modeling. J. Am. Ceram. Soc..

[27] Fan Z., Hsiao K.T., Advani S.G. (2004). Experimental investigation of dispersion during flow of multi-walled carbon nanotube/polymer suspension in fibrous porous media. Carbon.

[28] Fernberg S.P., Lundström T.S., Sandlund E.J. (2006). Mechanisms controlling particle distribution in infusion molded composites. J. Reinf. Plast. Compos..

[29] Ball R.C., Richmond P. (1980). Dynamics of colloidal dispersions. Phys. Chem. Liq..

[30] Krieger I.M., Dougherty T.J. (1959). A mechanism for non-Newtonian flow in suspensions of rigid spheres. Trans. Soc. Rheol. III.

[31] Patton T.C. (1964). Paint Flow and Pigment Dispersion.

[32] Barnes H.A., Hutton J.F., Walters K. (1989). An Introduction to Rheology.

[33] Gupta R.K., Advani S.G. (1994). Particulate suspensions. Flow and Rheology in Polymer Composites Manufacturing.

[34] Matijasic G., Glasnovic A. (2002). Influence of dispersed phase characteristics on rheological behavior of suspension. Chem. Biochem. Eng. Q..

[35] Rossi S., Luckham P.F., Tadros T.F. (2002). Influence of non-ionic polymers on the rheological behaviour of Na^+^-montmorillonite clay suspensions—I Nonylphenol—Polypropylene oxide—Polyethylene oxide copolymers. Colloids Surf. A.

[36] (2011). ASTM-D2584-11 Standard Test Method for Ignition Loss of Cured Reinforced Resins.

[37] Garay A.C., Heck V., Zattera A.J., Souza J.A., Amico S.C. (2011). Influence of calcium carbonate on RTM and RTM light processing and properties of molded composites. J. Reinf. Plast. Compos..

[38] Amico S., Lekakou C. (2004). Flow through a Two-Scale Porosity, Oriented Fibre Porous Medium. Transp. Porous Media.

[39] Sun X., Lasecki J., Zeng D., Gan Y., Su X., Tao J. (2015). Measurement and quantitative analysis of fiber orientation distribution in long fiber reinforced part by injection molding. Polym. Test..

[40] Di Giuseppe E., Castellani R., Dobosz S., Malvestio J., Berzin F., Beaugrand J., Delisée C., Vergnes B., Budtova T. (2016). Reliability evaluation of automated analysis, 2D scanner, and micro-tomography methods for measuring fiber dimensions in polymer-lignocellulosic fiber composites. Compos. Part A Appl. Sci. Manuf..

[41] Paes G. (2014). Fluorescent probes for exploring plant cell wall deconstruction: A review. Molecules.

[42] Gierlinger N., Schwanninger M. (2007). The potential of Raman microscopy and Raman imaging in plant research. J. Spectrosc..

[43] Delhaye M., Dhamelincourt P. (1975). Raman microprobe and microscope with laser excitation. J. Raman Spectrosc..

[44] Schaeberle M.D., Morris H.R., Turner J.F., Treado P.J. (1999). Raman Chemical Imaging Spectroscopy. Anal. Chem..

[45] Barbillat J., Turrel G., Corset J. (1996). Raman imaging. Raman Microscopy.

[46] Schmidt U., Mueller J., Weishaupt K., Hollricher O. (2008). Analysis of Multi-Component Polymer Blends with the Confocal Raman AFM. Microsc. Microanal..

[47] Gallos A., Paes G., Legland D., Allais F., Beaugrand J. (2017). Exploring the microstructure of natural fibre composites by confocal Raman imaging and image analysis. Compos. Part A Appl. Sci. Manuf..

[48] Shojaee S.A., Zandiatashbar A., Koratkar N., Lucca D.A. (2013). Raman spectroscopic imaging of graphene dispersion in polymer composites. Carbon.

[49] Rafiee M.A., Rafiee J., Srivastava I., Wang Z., Song H., Yu Z.-Z., Koratkar N. (2010). Fracture and fatigue in graphene nanocomposites. Small.

[50] Van Velthem P., Ballout W., Dumont D., Daoust D., Sclavons M., Cordenier F., Pardoen T., Devaux J., Bailly C. (2015). Phenoxy nanocomposite carriers for delivery of nanofillers in epoxy matrix for resin transfer molding (RTM)-manufactured composites. Compos. Part A Appl. Sci. Manuf..

